# Probiotic *Lactobacillus plantarum* GUANKE effectively alleviates allergic rhinitis symptoms by modulating functions of various cytokines and chemokines

**DOI:** 10.3389/fnut.2023.1291100

**Published:** 2024-01-15

**Authors:** Haijun Han, Guoliang Chen, Bin Zhang, Xuewen Zhang, Jingmin He, Wenjuan Du, Ming D. Li

**Affiliations:** ^1^Key Laboratory of Novel Targets and Drug Study for Neural Repair of Zhejiang Province, School of Medicine, Hangzhou City University, Hangzhou, China; ^2^State Key Laboratory for Diagnosis and Treatment of Infectious Diseases, National Clinical Research Center for Infectious Diseases, Collaborative Innovation Center for Diagnosis and Treatment of Infectious Diseases, The First Affiliated Hospital, Zhejiang University School of Medicine, Hangzhou, China; ^3^Department of Animal Science, Shanxi Agricultural University, Taigu, Shanxi, China; ^4^College of Biological Sciences, Shanxi Agricultural University, Taigu, Shanxi, China

**Keywords:** allergic rhinitis, probiotic, *Lactobacillus plantarum*, GUANKE, cytokine

## Abstract

**Background:**

Currently, the prevalence of allergic rhinitis (AR) remains high and there is a great need to develop better and safer ways to alleviate AR symptoms. The *Lactobacillus plantarum* GUANKE probiotic was reported as an immunomodulator through maintaining Th1/Th2 balance. This study aimed to determine the efficacy of GUANKE in AR subjects.

**Methods:**

Adults aged from 18 to 60 years old and previously suffered from AR were recruited and received GUANKE probiotics treatment for 4 weeks. The questionnaires of Total nasal symptom scores (TNSS), total non-nasal symptom score (TNNSS), and rhinitis control assessment test (RCAT) were used to assess the effectiveness before and after treatment. The serum allergen-specific IgE and cytokines were also determined at baseline and after 4 weeks of probiotics administration.

**Results:**

The results showed that TNSS and TNNSS were significantly reduced and the RCAT score was significantly increased compared to baseline. The sub-symptom score of rhinorrhea, itching, sneezing, and tearing in each questionnaire also showed significant changes, and the serum IgE level was markedly decreased. We further measured inflammatory-related proteins in serum and found that a total of 20 proteins (6 upregulated and 14 downregulated) were significantly changed compared to baseline, including IL-4, IL-7, IL-20, IL-33, CXCL1, CXCL5, CXCL6, CXCL11, CCL4, CCL23, TGF-alpha, LAP-TGF-beta-1, MMP-1, MMP-10, AXIN1, NT-3, OSM, SCF, CD6, and NRTN. Enrichment analysis showed that these significantly altered proteins were mainly enriched in cytokine and chemokine-related signaling pathways.

**Conclusion:**

Taken together, this study demonstrated the *Lactobacillus plantarum* GUANKE can serve as an effective immunobiotic for the treatment of AR, which is realized through maintaining the Th1/Th2 balance by modulating the functions of various cytokines and chemokines.

## Introduction

Allergic rhinitis (AR) is a non-infectious inflammatory disease of the nasal mucosa, which is characterized by stuffy nose, itchy nose, sneezing, and runny nose. AR is accompanied by eye burning, itchy eyes, pharyngeal congestion, tears and other eye symptoms, and also causes nasal polyps, sinusitis and other complications (1). In recent years, the prevalence of AR has shown a significant increase globally, affecting 10–20% population and becoming one of the most common chronic respiratory inflammatory diseases worldwide (2). According to the epidemiological surveys, the average prevalence of AR in Europe and North America is about 25%, while the AR incidence trend in both adults and children in China is still growing in recent years and has a significant effect on the general public (3).

The pathogenesis of AR is complex and is influenced by genotypic, epigenetic, and environmental factors (4). As a complex human disorder, its severity is commonly assessed by measures such as total nasal symptom scores (TNSS), total non-nasal symptom score (TNNSS), and rhinitis control assessment test (RCAT) (5). Of these measures, they generally include the severity of symptoms such as nasal congestion, rhinorrhea, nasal itching, sneezing, or other sub-symptoms or whether the rhinitis-related symptoms were improved or controlled. Its pathological mechanism is that after the susceptible individuals are exposed to allergen, non-infectious inflammatory diseases of the nasal mucosa are mainly mediated by immune globulins E (IgE) and induced by a variety of immune active cells and cytokines. The production of AR is closely related to the occurrence of T helper 2 (Th2) immune response, which is mainly manifested in the shift of the immune response of the body to various allergens to the Th2 type, that is, the Th1 type cell response is suppressed, and the Th2 type cell response is enhanced. The interaction of multiple cytokines secreted by Th1 and Th2 cells disrupts the balance, leading to imbalanced T lymphocyte differentiation and the release of a large amount of histamines (6, 7).

Currently, there are many kinds of drugs for AR treatment, such as antihistamines, intranasal corticosteroids, anticholinergics, antileukotrienes, to name a few, but long-term use of these drugs will bring undesirable side effects (8). In recent years, several studies have reported that probiotics are an alternative strategy for the treatment of AR (9–11). Probiotics can act as an immunomodulator to activate the host defense and regulate the immune response in the respiratory system (10). Some *Lactobacillus* species have been reported to have excellent immunomodulatory ability in respiratory diseases (12). The probiotic of GUANKE strain belongs to *Lactobacillus plantarum*, originally isolated from the fecal sample of a healthy individual, which was first found to be able to promote SARS-CoV-2 specific immune responses through enhancing interferon signaling and suppressing apoptotic and inflammatory pathways by acting as an immunomodulator role via maintaining Th1/Th2 balance (13). However, whether GUANKE probiotic can be a candidate immunobiotic for the treatment of AR remains to be determined, which formed the main purpose of this study.

## Materials and methods

### Subjects

A total of 47 adult subjects with perennial AR were recruited from local community clinics or health service centers in the city of Hangzhou area of Zhejiang province, China for this study. The inclusion criteria of each participant were: (1) diagnosed with rhinitis or symptoms; (2) aged between 18 and 60 years; (3) no other diagnosed diseases at the time of recruitment; (4) did not take any drugs for rhinitis or other immune-related diseases. The exclusion criteria of subjects included those who: (1) suffered from other definite respiratory or diseases; (2) received systemic steroids or antihistamines within 3 days; (3) were pregnant. All subjects provided written informed consent. The study protocol was approved by the Institutional Ethics Committee of the First Affiliated Hospital of Zhejiang University School of Medicine.

### Study product

The products used in the study is GUANKE Immunobiotics (Maiyata Inc., Shaoxing, Zhejiang, China), which is a probiotic mixture of GUANKE (*Lactobacillus plantarum*, CGMCC No. 21720) freeze-dried with maltodextrin, cranberry fruit powder, erythritol, isomaltulose and vitamin C. All study products were produced at a Good Manufacturing Practice-certified manufacturing facility.

### Study design

All recruited subjects were orally taken 2 packs of probiotics (1.5 g per stick pack contains 5.0 × 10^10^ CFU, Lot number: 22AR292) per day. Peripheral blood was collected and serum was isolated immediately before and after taking 4-week probiotics. The serum was stored at −80°C refrigerator for measuring IgE and cytokines. Three questionnaires were filled in by every participant at each visit.

### Total nasal symptom score

TNSS is one of the validated AR questionnaires (14), which is expressed as the sum of the scores for four symptoms (nasal congestion, rhinorrhea, nasal itching, and sneezing). Each symptom was rated on a 4-point scale from 0 (no symptoms), 1 (mild symptoms), 2 (moderate symptoms), to 3 (severe symptoms), and recorded at 0 week and 4 weeks after taking probiotics. The sum score of each symptom was used to evaluate rhinitis-related symptoms before and after treatment with the probiotic.

### Total non-nasal symptom score

TNNSS is the second questionnaire used in this study (14), which is based on the presence or absence of symptoms, such as post-nasal discharge, tearing, nasal or ocular itching, nasal or maxillary pain, and headache. Each item was assigned to two grades: symptomatic and asymptomatic. If no symptom occurs, the score is 0 point, otherwise the score is 1 point for each abovementioned symptom. The total score ranges from 0 to 5. The sum score of each symptom was used to evaluate non-rhinitis symptoms before and after treatment.

### Rhinitis control assessment test

RCAT is the third questionnaire used in the study (15), which consists of six items: nasal congestion, sneezing, watery eyes, sleep interference, daily activities, and degree of rhinitis control. Each item is assigned 1–5 points, and the score of each item are summed up for evaluating the level of rhinitis control. A higher score indicates better rhinitis control.

### Measurement of IgE in serum

Serum IgE level was determined by ELISA (E-EL-H6104, Elabscience Biotechnology Co., Ltd., Wuhan, China) according to the manufacturer’s introduction. Briefly, a total of 100 μL standard or serum samples were added into the corresponding well, and the plate were coated and incubated at 37°C for 90 min. After draining each well, 100 μL Biotinized antibody working solution was added into each well and incubated at 37°C for 1 h with enzymic labeled plate and film coating. Then, 350 μL washing liquid was added into each well, incubated for 1–2 min, then discarded liquid and dried on a paper. After repeating this step for three times, 100 μL enzyme conjugate working solution was then added into each well, and incubated at 37°C for 30 min before draining the liquid and washing the plate for five times. Then, 90 μL substrate solution (TMB) was added to each well, and the plate was coated and incubated at 37°C for 15 min in the dark. Finally, 50 μL termination solution was added to each well to terminate the reaction. The optical density (OD value) of each well was immediately measured with the enzyme label instrument at the wavelength of 450 nm. The concentration of human IgE in each sample was calculated by comparing the OD of the samples to the standard curve.

### Determination of inflammatory cytokines in serum

Inflammatory cytokines were measured using the Olink^®^ Target 96 Inflammation Panel (Olink Proteomics AB, Uppsala, Sweden) according to the manufacturer’s instructions. This panel enables 92 cytokines to be analyzed simultaneously by using 1 μL of each sample. In brief, pairs of oligonucleotide-labeled antibody probes bind to their targeted protein, and if the two probes are brought in close proximity the oligonucleotides will hybridize in a pair-wise manner. The addition of a DNA polymerase leads to a proximity-dependent DNA polymerization event, generating a unique PCR target sequence. The resulting DNA sequence is subsequently detected and quantified using a microfluidic real-time PCR instrument (Signature Q100, LC-Bio Technology Co., Ltd., Hangzhou, China). The resulting Ct-data is then quality-controlled and normalized using a set of internal and external controls. The final assay read-out is presented in Normalized Protein expression (NPX) values, which is an arbitrary unit on a log2-scale where a high value corresponds to a higher protein expression.

### Statistical analysis

The normality was performed using the Kolmogorov–Smirnov test. If the data did not distribute normally, the difference was determined by the Wilcoxon matched-pairs *t*-test of the results before and after the treatment. All values are expressed as mean ± standard error of the mean (SEM). Statistical analysis was performed using GraphPad Prism v.8.0 Software (GraphPad Inc., San Diego, CA, United States), and the *p* < 0.05 was defined as statistical significance.

## Results

### Effects of GUANKE probiotic on rhinitis symptoms

**Figure 1:**
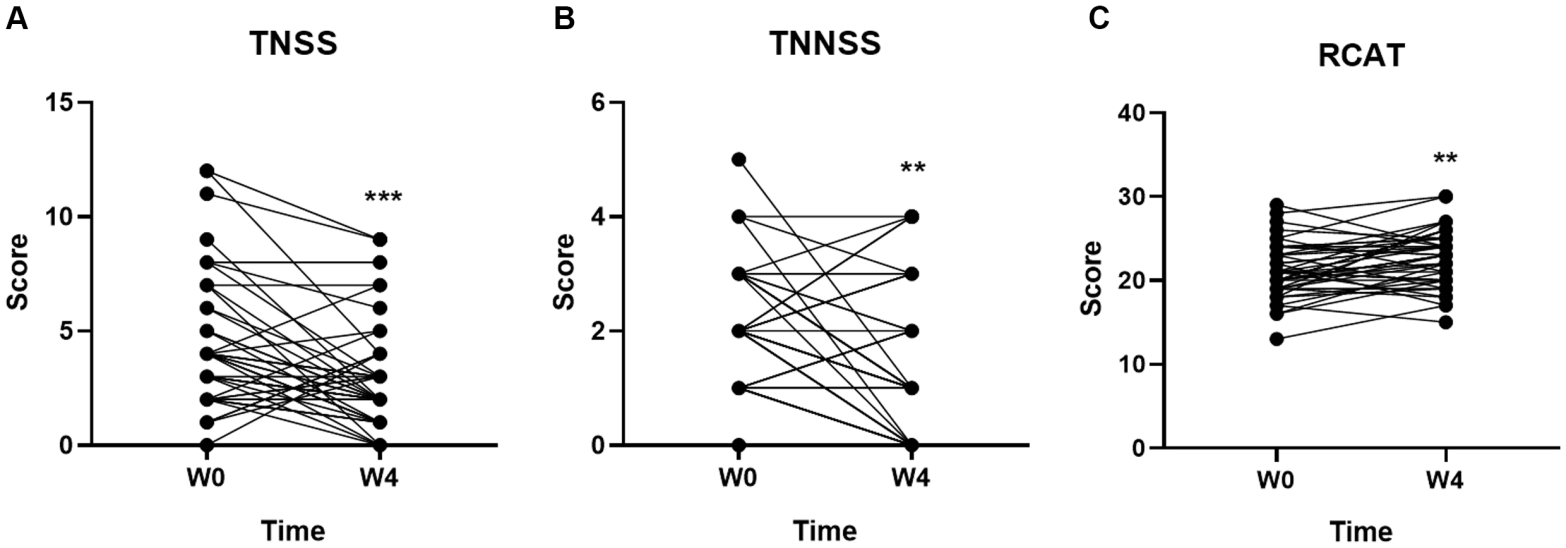
Effect of GUANKE probiotic on AR symptoms evaluated by three questionnaires **(A)** TNSS, **(B)** TNNSS, and **(C)** RCAT. The scores are presented as the sum of the all symptoms in each questionnaire. The significance was evaluated by performing paired *t*-test. *Indicates a statistically significant difference between the different time points. **p < 0.01, ***p < 0.001.

Compared with the baseline (Week 0), both the TNSS (Figure 1A) and TNNSS (Figure 1B) were significantly reduced after 4-week probiotic treatment (*p* < 0.001 for both). Further, the total score of RCAT (Figure 1C) was markedly increased after taking 4-week probiotic (*p* < 0.01), indicating the rhinitis symptoms were well controlled by GUANKE probiotic.

### Changes of the symptoms in TNSS from baseline

**Figure 2:**
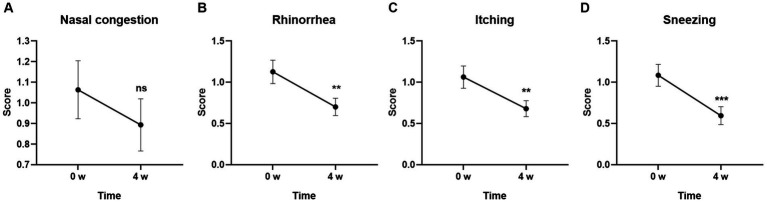
Effect of GUANKE probiotic on AR related sub-symptoms in TNSS questionnaire. **(A)** Nasal congestion, **(B)** rhinorrhea, **(C)** itching, **(D)** sneezing. The score of each sub-symptom was presented. The significance was evaluated by performing paired *t*-test. *Indicates a statistically significant difference between the different time points. **p < 0.01, ***p < 0.001, ns indicates no statistical significance.

To further characterize the individual symptom alterations in each questionnaire, we first compared the individual symptoms in TNSS. As shown in Figure 2, three of four symptoms including Rhinorrhea (*p* < 0.01), Itching (*p* < 0.01), and Sneezing (*p* < 0.001) were significantly changed after 4-week GUANKE probiotic treatment. Although the Nasal congestion score did not obtain significance (Figure 2A), it also showed a decrease trend at Week 4.

### Changes of the symptoms in TNNSS from baseline

**Figure 3:**
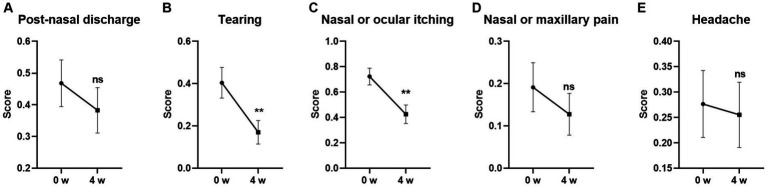
Effect of GUANKE probiotic on AR related sub-symptoms in TNNSS questionnaire. **(A)** Post-nasal discharge, **(B)** tearing, **(C)** nasal or ocular itching, **(D)** nasal or maxillary pain, **(E)** headache. The score of each sub-symptom was presented. The significance was evaluated by performing paired *t*-test. *Indicates a statistically significant difference between the different time points. **p < 0.01, ns indicates no statistical significance.

Among the five individual symptoms in TNNSS, the scores for Tearing and Nasal or Ocular itching were significantly decreased from baseline after 4-week GUANKE probiotic treatment (Figures 3B,C) (*p* < 0.01 for each). The other three symptoms including Post-nasal discharge, Nasal or maxillary pain, and Headache also showed a decreased trend compared to the baseline, however, no statistical significance was detected (Figures 3A,D,E) (*p* > 0.05 for each).

### Changes of the symptoms in RCAT from baseline

**Figure 4:**
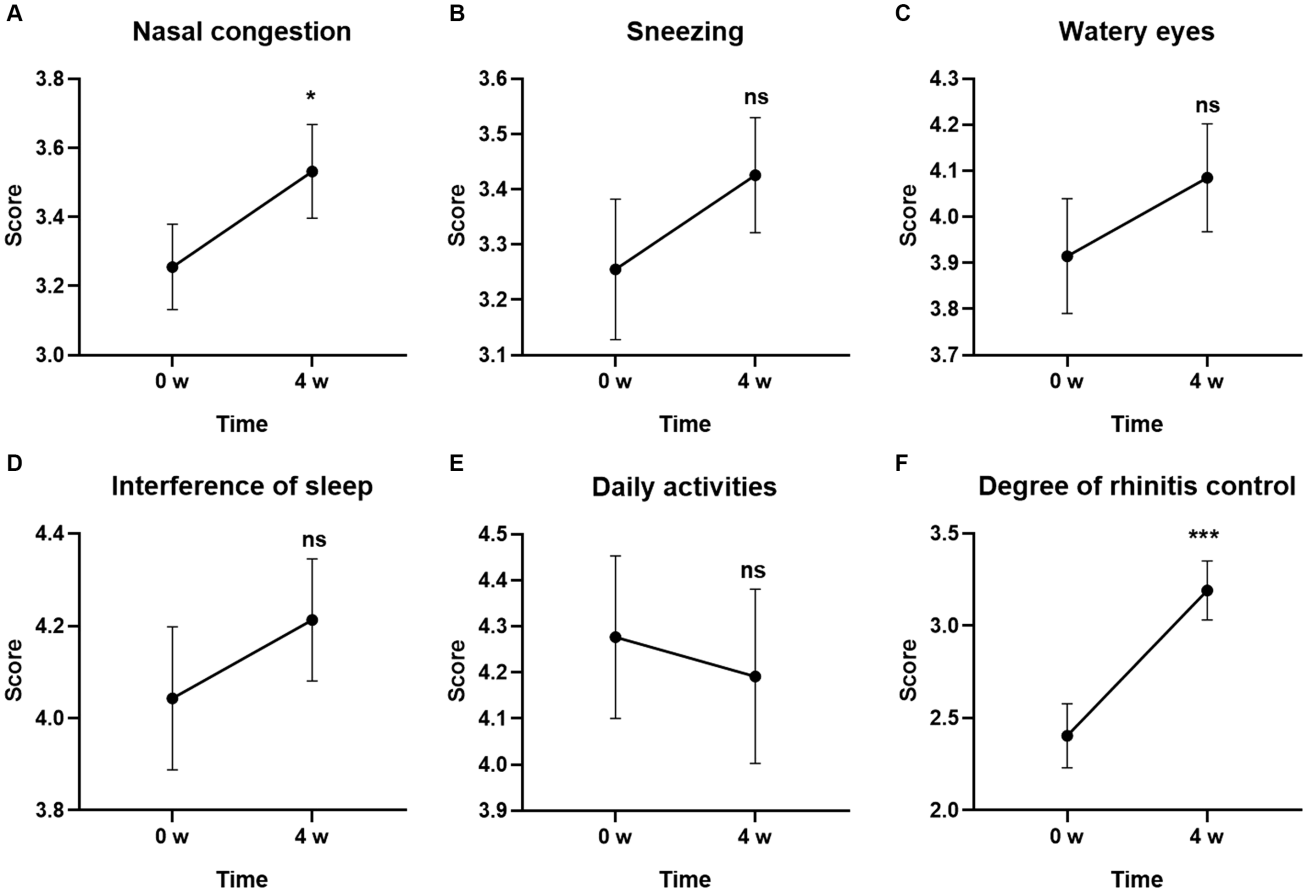
Effect of GUANKE probiotic on AR related sub-symptoms in RCAT questionnaire. **(A)** Nasal congestion, **(B)** sneezing, **(C)** watery eyes, **(D)** interference of sleep, **(E)** daily activities, **(F)** degree of rhinitis control. The score of each sub-symptom was presented. The significance was evaluated by performing paired *t*-test. *Indicates a statistically significant difference between the different time points. *p < 0.05, ***p < 0.001, ns indicates no statistical significance.

Among the individual symptoms in RCAT, the score for Degree of rhinitis control was the most obvious improvement at Week 4. GUANKE probiotic treatment significantly controlled rhinitis symptoms compared to the baseline (Figure 4F) (*p* < 0.001). Nasal congestion was also significantly improved after 4-week treatment (Figure 4A) (*p* < 0.05). The other scores including Sneezing, Watery eyes, Interference of sleep, and Daily activities tended to be different between the week 0 and after 4-week of treatment but did not reach statistical significance (Figures 4B–E) (*p* > 0.05 for each).

### Effects of GUANKE probiotic on serum IgE concentration

**Figure 5:**
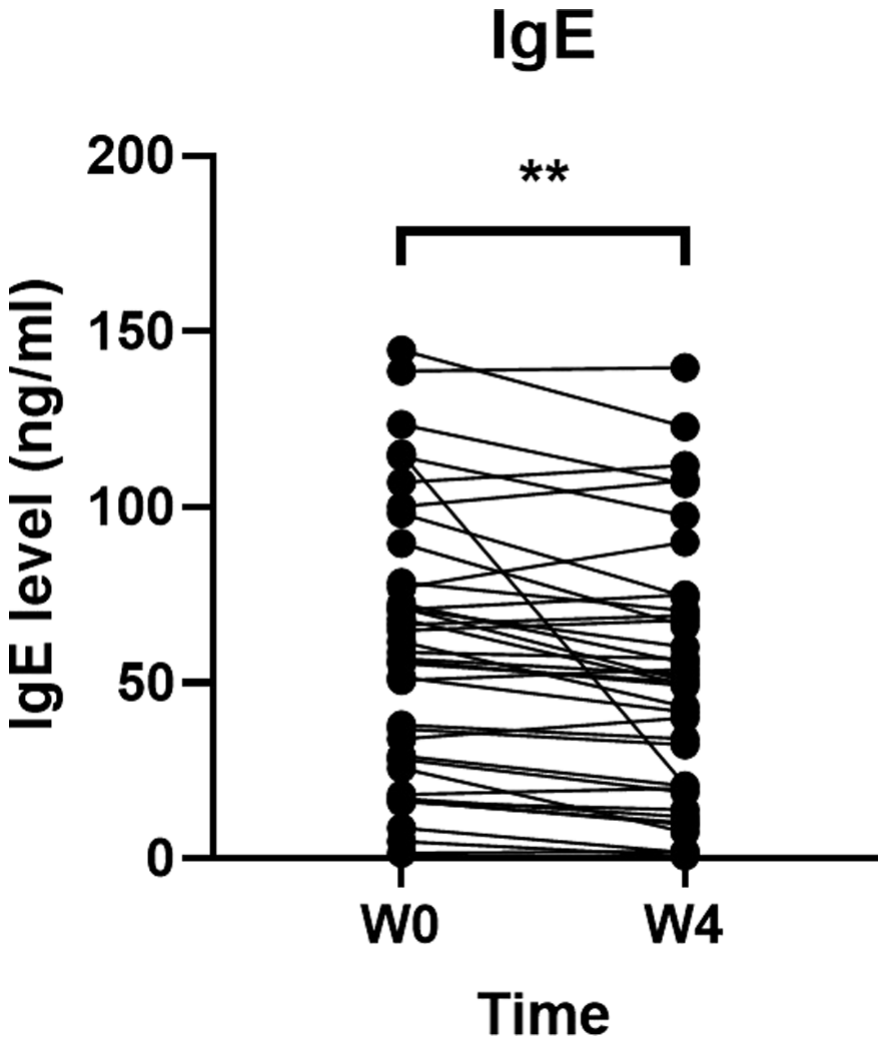
Effect of GUANKE probiotic on IgE level before (Week 0, baseline) and after (4 weeks) treatment. The significance was evaluated by performing paired *t*-test. *Indicates a statistically significant difference between the different time points. **p < 0.01.

To further investigate the effects of GUANKE probiotic on allergic rhinitis, we determined the IgE concentration in serum. As shown in Figure 5, the level of IgE was significantly decreased after 4-week GUANKE treatment (*p* < 0.01), indicating the improvement of GUANKE on AR symptoms was indeed mediated by IgE.

### Effects of GUANKE probiotic on serum cytokines level

**Figure 6:**
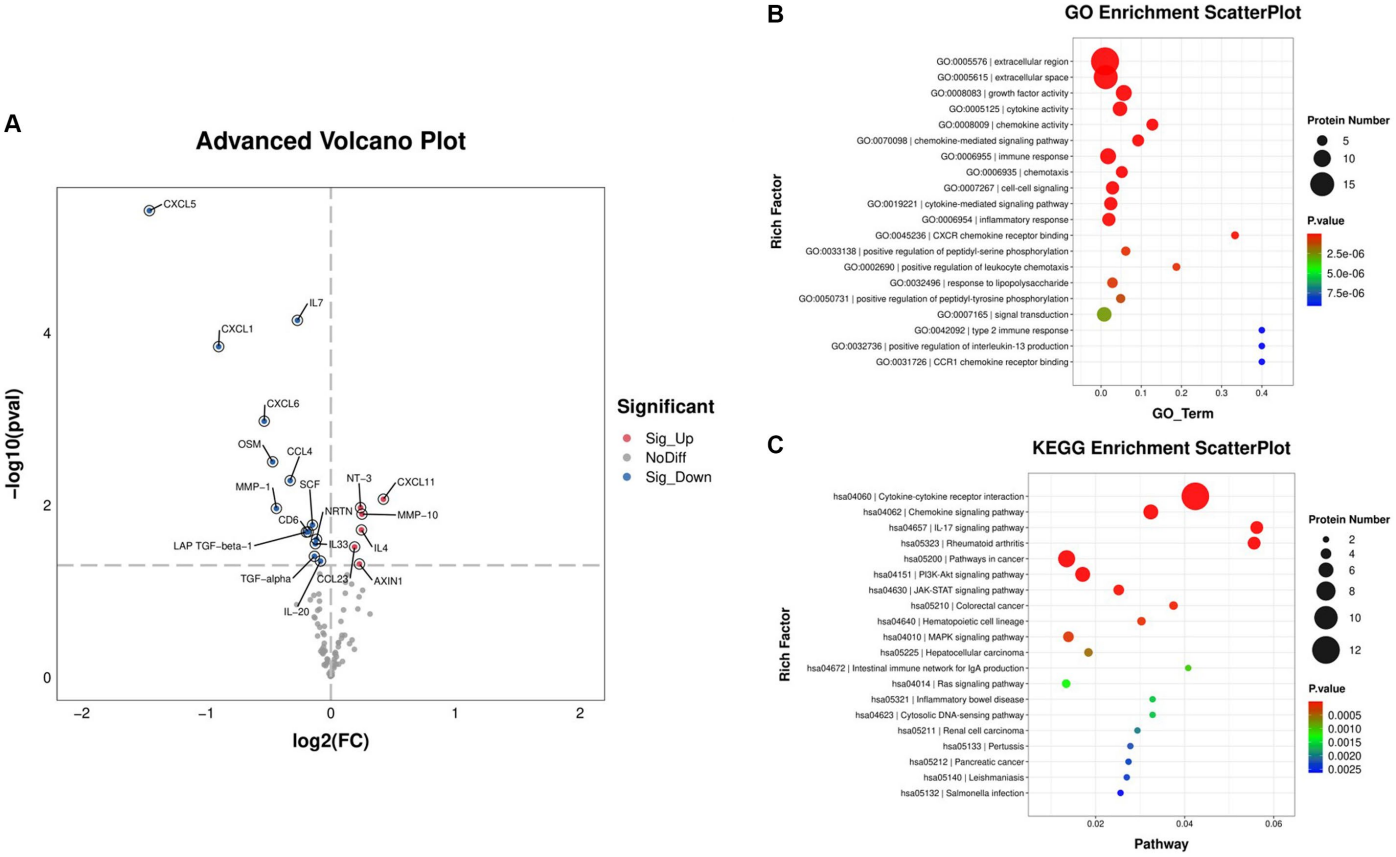
Differentially expressed cytokines before (week 0, baseline) and after 4 weeks of GUANKE treatment. **(A)** Volcano plot of the distribution of differentially expressed proteins. Red dots: significantly upregulated proteins, blue dots: significantly downregulated proteins, gray dots: non-significantly expressed proteins. The threshold is *p* < 0.05, and fold change >2. Scatter plot of **(B)** GO and **(C)** KEGG enrichment analysis for the above differentially expressed proteins. The color indicates the different *p*-value. The dot size represents the number of proteins.

Through performing inflammatory-related proteins determination by Olink, we found that a total of 20 proteins were significantly changed after 4-week GUANKE probiotic treatment (Supplementary Figure S1), which include six significantly upregulated proteins: NT-3, CXCL11, MMP-10, IL-4, CCL23, and AXIN1, and 14 significantly downregulated proteins: CXCL5, IL-7, CXCL1, CXCL6, OSM, CCL4, MMP-1, SCF, CD6, NRTN, LAP-TGF-beta-1, IL-33, TGF-alpha, and IL-20 (Figure 6A; *p* < 0.05). We further performed GO enrichment analysis (Figure 6B), which showed that most of these markedly changed proteins were enriched in extracellular region and space. Functionally, these proteins mainly enriched in growth factor activity, cytokine activity, and chemokine activity, as well as chemokine and cytokine-mediated signaling pathways. Further, KEGG enrichment analysis also indicated these proteins were enriched in cytokine and chemokine-related signaling pathways and function (Figure 6C). Taken together, this suggests that GUANKE probiotic mainly improves symptoms of AR through mediating cytokines and chemokines to maintain Th1/Th2 balance.

## Discussion

In the present study, we demonstrated the efficacy of GUANKE probiotic on the improvement of allergic rhinitis. Our results clearly indicated that GUANKE probiotic can significantly improve the symptoms of allergic rhinitis, such as rhinorrhea, itching, sneezing, and tearing, which are mainly modulated by IgE and inflammatory cytokines and chemokines.

Recently, a number of studies have reported that probiotics can serve as an alternative method in treating AR (16, 17). Both human and animal studies have demonstrated various probiotics could alleviate AR symptoms and quality of life (10). Anania et al. (18) found that a mixture of *Bifidobacterium animalis* subsp. *Lactis* BB12 and *Enterococcus faecium* L3 probiotics could reduce symptoms of AR in children. Kang et al. (19) reported that 4 weeks treatment with probiotic NVP-1703 (a mixture of *Bifidobacterium longum* and *Lactobacillus plantarum*) alleviated perennial AR mediated by IgE and IL-10. Torre et al. (20) used iPROB^®^ probiotic preparation (Anallergo SpA, Florence, Italy) to treat AR patients for 60 days and found a significant decrease in Average Rhinitis Total Symptom Score and improvement in quality of life. In animal studies, Choi et al. (21) found that oral administration of *Lactobacillus plantarum* (CJLP133 and CJLP243) improved the symptoms and reduced the inflammation in a birch pollen-induced AR mouse model. Similarly, oral administration of another *Lactiplantibacillus plantarum* NR16 reduced airway hyperresponsiveness and leukocyte infiltration in lesions of birch pollen-induced AR mice (22). Lin et al. (23) applied *Lacticaseibacillus paracasei* GM-080 in both ovalbumin (OVA)-induced AR mouse model and perennial AR children, and the results indicated that it significantly ameliorates allergic airway inflammation. Here, we are the first to apply *Lactobacillus plantarum* GUANKE probiotic in AR. In line with the above studies, we found that 4 weeks of GUANKE probiotic treatment can also effectively control the AR symptoms such as rhinorrhea, itching, sneezing, and tearing, demonstrating its efficacy in the improvement of AR.

Allergic rhinitis triggers a systemic increase of inflammation with various cytokines release (24). Recently, a large number of inflammatory cytokines have been found significantly changed in AR, although the molecules regulated by different probiotics appeared to vary (25). In the present results, GUANKE probiotic mainly regulated the levels of inflammatory cytokines, such as IL-4, IL-7, IL-20, and IL-33; chemokines, such as CXCL1, CXCL5, CXCL6, CXCL11, CCL4, and CCL23; as well as other cytokines, such as TGF-alpha, LAP-TGF-beta-1, MMP-1, MMP-10, AXIN1, NT-3, OSM, SCF, CD6, and NRTN. These significantly altered molecules were mainly enriched in cytokine and chemokine-related signaling pathways and corresponding functions.

The inflammatory cytokines are the main regulated molecules involved in AR. IL-4 plays a key role in inducing IgE production and acts as a therapeutic target in AR (26). In this study, we found that IL-4 level was significantly increased after GUANKE probiotic treatment. Although it was in contrast with the previous reports as a Th2 cytokine, here, we speculated it acted as an anti-inflammatory cytokine (27). IL-10 is another well-known anti-inflammatory cytokine, which also showed an increased trend after treatment although it did not obtain statistical significance, suggesting that GUANKE probiotic enhanced anti-inflammatory ability. IL-7 is of great significance in regulating the immune function of the body, which has been demonstrated to be necessary for the generation and maintenance of T and B cells, and lack of IL-7 would cause immature immune cell arrest (28, 29). IL-20 is a pro-inflammatory mediator, which can regulate cytokine and chemokine expression in different types of cells (30). Recent studies have shown that IL-20 plays an important role in the pathogenesis of bronchial asthma (31). It participates in T cell-mediated disease development by regulating cytokines secreted by T cells. Long-term exposure of T cells to IL-20 can cause an increase in initial T cell polarization toward Th2 cells, resulting in increased secretion of IL-4 and IL-13, and reduced secretion of IFN-γ (32) and participating in the occurrence of AR by affecting the Th1/Th2 balance. IL-33 plays a pro-inflammatory role in allergic diseases and is recognized as an important contributor to Th2-type immune responses (33). The elevated level of IL-33 in serum correlated significantly with the severe symptoms of AR patients (34), and immunotherapy can decrease IL-33 levels and improve AR symptoms (35). Here, we also found the dysregulation of these inflammatory cytokines after GUANKE probiotic administration for 4 weeks, suggesting GUANKE probiotic in AR treatment mainly functioned as an immunomodulator.

Except for inflammatory cytokines, the chemokines and chemokine receptors also play critical roles in AR pathogenesis. They participate in all of the three phases of AR by promoting inflammatory cell recruitment, differentiation, and allergic mediator release, and also be therapeutic targets for AR (36). The origin of chemokines and the inflammatory cells are different, but CCL and CXCL are two types of chemokines that are more closely related to the development of AR (37). Here, we found that 6 chemokines were significantly changed after GUANKE probiotic treatment. CXCL1, CXCL5, and CXCL6 were markedly decreased, which receptor is CXCR2, expressed in neutrophils, monocytes, NK cells, mast cells, and basophils, and the main function is related to B cell lymphopoiesis and neutrophil trafficking. While CXCL11 was significantly increased, which receptor is CXCR3, expressed in Th1 cells, CD8+ T cells, and NK cells, and the main function is related to Type I adaptive immunity (37). Previous studies also showed that CCL4 and CCL23 were also significantly changed in AR patients (38, 39). Taken together, these data demonstrated the importance of chemokines in AR.

In recent years, the regulatory role of probiotics in allergic rhinitis has expanded beyond the Th1/Th2 balance to other T cell subsets, such as regulatory T (Treg) cells. Foxp3 is a key regulatory factor for the normal development and function of Treg cells (40). *Lactobacillus rhamnosus* has been shown to maintain the Treg/Th2 balance by increasing the levels of Foxp3 + Treg cells (41). Additionally, spore-forming *Bacillus* species can induce Foxp3 + Treg cells to suppress inflammatory reactions (42). IL-10 is also a crucial anti-inflammatory factor, and probiotics can induce IL-10 production, promoting the differentiation of Treg cells (43–45). Furthermore, short-chain fatty acids (SCFAs), as major metabolites of intestinal microbiota fermentation, have been reported to regulate immune responses in multiple organs and maintain intestinal homeostasis (46). Gu et al. found a significant upregulation of SCFAs levels and a reduction in inflammation in a mouse model following gavage with *Lactobacillus plantarum* ZJ316 (47). Studies have shown that an increased abundance of microbiota-producing SCFAs in the gut can enhance the generation of Treg cells (48). Further research is needed to investigate whether the GUANKE strain affects allergic rhinitis symptoms through the regulation of these pathways.

Finally, the potential limitations of this study we want to address. First, the sample size was relatively small. Despite of this, we still observed significant changes of symptoms and cytokines before and after GUANKE probiotic treatment in AR patients. Second, we only compared two time points that were before and after 4-week GUANKE probiotic administration. It would be better to compare more time points such as shorter or longer treatment, which could characterize the process of dynamic changes after GUANKE treatment. Third, the randomized, double-blind, placebo-controlled study is better to demonstrate the efficacy of GUANKE probiotic in AR treatment, and this study is currently ongoing.

In conclusion, we provide a new probiotic *Lactobacillus plantarum* GUANKE for the improvement of AR. Our findings revealed that the GUANKE can effectively alleviate not only the symptoms of AR but also maintain the Th1/Th2 balance along with IgE, cytokines, and chemokines alterations.

## Data availability statement

The raw data supporting the conclusions of this article will be made available by the authors, without undue reservation.

## Ethics statement

The studies involving humans were approved by the Institutional Ethics Committee of the First Affiliated Hospital of Zhejiang University School of Medicine. The studies were conducted in accordance with the local legislation and institutional requirements. The participants provided their written informed consent to participate in this study.

## Author contributions

HH: Project administration, Writing – review & editing, Conceptualization, Data curation, Investigation, Methodology, Writing – original draft. GC: Data curation, Investigation, Methodology, Writing – original draft. BZ: Writing – review & editing. XZ: Data curation, Investigation, Writing – original draft. JH: Writing – original draft, Data curation, Investigation, Writing – review & editing. WD: Data curation, Investigation, Writing – review & editing. MDL: Funding acquisition, Project administration, Supervision, Writing – review & editing, Conceptualization, Investigation, Resources.
