# Supplementary material for: Probiotic Lactobacillus plantarum GUANKE effectively alleviates allergic rhinitis symptoms by modulating functions of various cytokines and chemokines
Source: Front Nutr. 2024 Jan 15;10:1291100. doi: 10.3389/fnut.2023.1291100 (PMC10822906; doi:10.3389/fnut.2023.1291100)
Supplement: Supplementary file 1 [file Table_1.DOCX]

**Table S1** Characteristics of the samples in the present study at baseline.

| **Characteristics** | **Sample information (n =47)** |
| --- | --- |
| Male (%) | 25.5 |
| Age, years [mean (SD)] | 36.4 (7.2) |
| Smoking rate (%) | 10.6 |
| Allergic rhinitis duration, years [mean (SD)] | 1.5 (1.0) |
| Symptom scores at baseline [mean (SD)] |  |
| TNSS | 4.3 (2.8) |
| TNNSS | 2.1 (1.1) |
| RCAT | 21.1 (3.2) |

Data are presented as mean (SD). TNSS, Total nasal symptom scores; TNNSS, total non-nasal symptom score; RCAT, rhinitis control assessment test.
