# Supplementary figures and images for: Probiotic Lactobacillus plantarum GUANKE effectively alleviates allergic rhinitis symptoms by modulating functions of various cytokines and chemokines
Source: Front Nutr. 2024 Jan 15;10:1291100. doi: 10.3389/fnut.2023.1291100 (PMC10822906; doi:10.3389/fnut.2023.1291100)

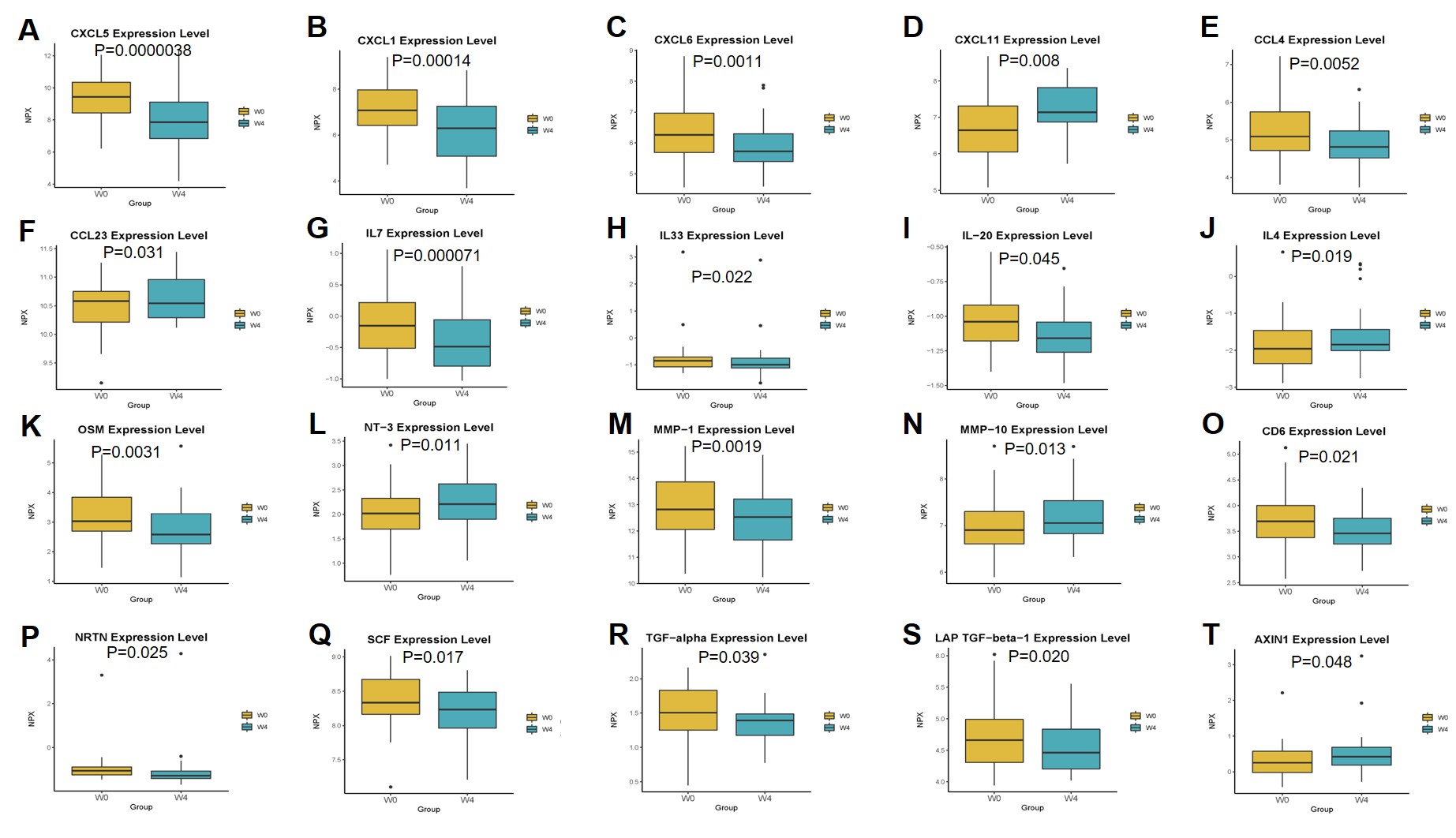

Supplement: Supplementary file 2 [file Image_1.JPEG]
